# Effects of Deep Electroacupuncture Stimulation at “Huantiao” (GB 30) on Expression of Apoptosis-Related Factors in Rats with Acute Sciatic Nerve Injury

**DOI:** 10.1155/2015/157897

**Published:** 2015-06-17

**Authors:** Lili Dai, Yanjing Han, Tieming Ma, Yuli Liu, Lu Ren, Zenghua Bai, Ye Li

**Affiliations:** ^1^Liaoning University of Traditional Chinese Medicine, Shenyang 110847, China; ^2^Liaoning Health Vocational and Technical College, Shenyang 110101, China; ^3^Institute of Acupuncture and Moxibustion, China Academy of Chinese Medical Sciences, Beijing 100700, China; ^4^Liaoning University of Traditional Chinese Medicine, Benxi Campus, Benxi 117000, China

## Abstract

SD rats were randomly divided into normal control, model, deep EA, and shallow EA groups. The model was established by mechanical clamping of the sciatic nerve stem. For deep and shallow EA, the needles were inserted into “Huantiao” (GB 30) by about 16 mm and 7 mm, respectively, once daily for 14 days. The results showed that, compared with the normal control group, the nerve-muscle excitability of rat's hip muscle decreased and the nerve conduction velocity of sciatic nerve slowed down in the model group; meanwhile, the number of apoptotic cells and the expression level of Bax protein in the injured nerve increased significantly, and the expression level of Bcl-2 protein and the ratio of Bcl-2/Bax decreased considerably. Compared with the model group, the indices mentioned above were reversed in the two treatment groups, and the changes in the deep EA group were more significant than those in the shallow EA group. These results indicate that EA stimulation at GB 30 can improve the function of injured sciatic nerve, which is closely associated with its effects in upregulating the expression of apoptosis inhibitive factor Bcl-2 and downregulating apoptosis promotive factor Bax. Deep EA is relatively better.

## 1. Introduction

Peripheral nerve injury is one of the most common traumatic disorders. Slow recovery and prolonged loss of sensation or motor function may cause muscle atrophy, joint contracture, and deformity. If the injured nerve fibers are repaired, the continuity of nerves can be restored, providing favorable conditions for nerve regeneration.

Acupuncture has been proven to be an effective method for the treatment of peripheral nerve injury and is widely used to promote the recovery of nerve function [1, 2]. Research has shown that the nerve stump is ischemic in the early stage and that local blood circulation can be improved by acupuncture therapy [3]. With regard to the related mechanism, research has proved the positive effect of acupuncture on the repair of injured nerves from the perspectives of behavior, electrophysiology, and morphology [4–7]. Clinical practice confirmed that the sense of electric shock induced by deep acupuncture at Huantiao (GB 30) had a significant effect on the functional recovery of injured nerves [8, 9]. Experimental studies showed that acupuncture at GB 30 had a positive influence on motor recovery [4] and facilitated axonal regeneration in the injured peripheral nerves [10]. Previous work by our research group indicated that deep electroacupuncture (EA) stimulation at GB 30 improved the pathological changes and function of the injured sciatic nerve in rats, which was closely associated with its effects on the upregulation of nerve growth factor expression and downregulation of Fos expression in the damaged sciatic nerve. Deep EA was found to be better than shallow EA [11]. The present study aimed to further investigate the mechanism of repair of sciatic nerve injury following acupuncture at GB 30 and the difference between deep EA and shallow EA in terms of apoptosis.

## 2. Materials and Methods

### 2.1. Animals and Grouping

This experiment was conducted in accordance with* the Guide for Care and Use of Laboratory Animals* issued by the National Institutes of Health.

Forty-eight healthy pathogen-free Sprague Dawley (SD) rats (24 male and 24 female) with a body mass of 250 ± 20 g, were provided by the Experimental Animal Center, Liaoning University of Traditional Chinese Medicine, license number: SCXK (Liaoning) 2008-0005. The laboratory environment was as follows: temperature was 18–22°C, indoor light exposure was approximately 8 h, and relative humidity was about 45%. Free access to water and food was allowed, and males and females were kept separately with six rats per cage. According to the random number table, the rats were randomly divided into the normal control, model, deep EA, and shallow EA groups with 12 in each group.

### 2.2. Main Reagents and Instruments

The following reagents and instruments were used: cell apoptosis detection kit (Roche), Bcl-2 (B-cell lymphoma/leukemia-2), and Bax (Bcl-2 associated X protein) immunohistochemical detection kits (Shanghai BlueGene Biotech Co., Ltd.), Hwato acupuncture needles (Suzhou Acupuncture Supplies Factory), pulse electroacupuncture therapeutic apparatus (6805-A, Shantou Medical Equipment Factory), biophysiological experimental system (BL-420, Chengdu Taimeng Electronics Co., Ltd.), microtome (RM 2235, Leica), digital microscope (BX 41, Olympus), and MetaMorph microscopic image analysis system (UIC, Olympus).

### 2.3. Modeling

The acute sciatic nerve injury model was established by mechanical clamping of the sciatic nerve stem [12]. The rat was placed in the prone position on the operating table, and anesthesia was given by intraperitoneal injection of 1% pentobarbital sodium (40 mg/kg). Routine skin preparation and sterilization were carried out, and a 1 cm vertical incision was made at the rear of the middle femoral shaft on the left to expose the bicep femoris muscle. The sciatic nerve was then dissociated by blunt dissection and then clamped with a 16 cm needle holder 0.5 cm below the femoral tubercle. The holder was released after squeezing for 10 s. This was repeated 3 times with an interval of 10 s until the sciatic nerve was seriously injured. The sciatic nerve with the injured trunk of about 3 mm was marked by a 9-0 noninvasive suture thread and then put back in place, and the skin was sutured. The above operation was performed by one person.

### 2.4. Processing Methods for Each Group

*Normal Control Group.* The rats were kept under the same conditions without modeling and treatment.

*Model Group.* The rats were kept under the same conditions after modeling without any treatment.

*Deep EA Group.* The sciatic nerve injury model was achieved by mechanical clamping of the sciatic nerve stem. After successful modeling, acupuncture was applied to the GB 30 (the depression in front of the femoral greater trochanter at the leading edge of the hip joint in the affected side) with the depth of about 16 mm to the extent that the muscle twitched instantly and the toes trembled. The electroacupuncture therapeutic apparatus was then connected, while the indifferent electrode was placed in the homolateral lower limb. Dilatational wave was applied with a frequency of 2 Hz/100 Hz and an intensity of about 2 mA and the affected limb was observed to twitch slightly. The treatment was given for 20 min each time, once daily for 14 days.

*Shallow EA Group.* The sciatic nerve injury model was achieved by mechanical clamping of the sciatic nerve stem. After successful modeling, acupuncture was applied to the GB 30 with the depth of about 7 mm without touching the nerve trunk. The electroacupuncture therapeutic apparatus was then connected, while the indifferent electrode was placed in the homolateral lower limb. Dilatational wave was applied with a frequency of 2 Hz/100 Hz and an intensity of about 2 mA and the affected limb was observed to twitch slightly. The treatment was given for 20 min each time, once daily for 14 days.

### 2.5. Criteria of Successful Modeling

Half an hour after modeling, 5 rats were randomly selected for EMG testing to observe the motor conduction velocity (MCV) of sciatic nerves. When the MCV dropped below 10 m/s, it was deemed that the sciatic neuraxon and myelin sheath had broken or were severely injured [13].

### 2.6. Observation Indices and Methods

*General Status.* The rats' mental state, limb activity, reaction, ingestion, water intake, and daily activities were assessed.

*Determination of the Strength-Duration (S-D) Curve.* The S-D curve was determined by applying the biophysiological experimental system to display the nerve-muscle excitability in the rat. The working electrode was placed on the buttock, and the auxiliary electrode was placed on the ankle joint of the homolateral posterior limb. First, the motor point of the muscle was detected by strong stimulation, and then the magnitude of the current was turned down when weak muscle twitches were observed with the naked eye. The stimulus threshold was measured with a pulse width of 0.1–1 ms, and the S-D curve was drawn using logarithmic coordinates.

*Detection of Conduction Velocity of the Sciatic Nerve.* The rat was placed in the prone position on the operating table, and anesthesia was given by intraperitoneal injection of 10% chloral hydrate (0.35 mg/100 g). The skin and muscles were cut using the modeling method to fully expose the sciatic nerve segment for surgery. The sciatic nerve was dissociated using a glass dissecting needle with two insulated bipolar acicular electrodes hooked at both ends of the nerve anastomosis, and the recording electrode was placed at the distal end of the stimulation electrode. The stimulus was then applied to determine the threshold causing evoked action potential. This stimulus was repeated until the graph of action potential on the screen remained stable and the starting points of the artifact and action potential became clear. Screenshots were then obtained, and the amplitude of action potential of the nerve trunk was automatically displayed. If the amplitude of action potential of the nerve trunk was measured from the highest point to the lowest point, the measurement of the latent period lasted from the appearance of stimulus artifact to the initiation site of action potential. When the distance between the two stimulation electrodes was input, the nerve conduction velocity was automatically displayed. During the operation, the sciatic nerves were covered with saline-soaked gauze to ensure that the exposed nerves and muscles remained moist.

*Detection of Cell Apoptosis by Terminal Deoxynucleotidyltransferase-Mediated dUTP-Biotin Nick-End Labeling (TUNEL).* 8 rats randomly selected in each group were perfused with 4% paraformaldehyde and fixed on the operating table. The injured sciatic nerve tissues were removed and fixed for 24 h. Routine paraffin sections (5 *μ*m) were prepared. After being dewaxed, the sections were treated according to the TUNEL kit instructions. Two sections were taken out from each rat. Eight nonoverlapped views were randomly selected for each section. The MetaMorph microscopic image analysis system was applied. Brown yellow particles in the nucleus were positive cells, and the number of apoptotic cells was measured.

*Determination of the Expression of Bcl-2 and Bax Protein by Immunohistochemistry.* After being dewaxed, the sections were treated with streptavidin-peroxidase (SP) immunohistochemistry. Three sections were taken out from each rat. Five no-overlapped views were randomly selected for each section. The MetaMorph microscopic image analysis system was used to measure the mean optical density of the positive products of Bcl-2 and Bax. The ratio of Bcl-2/Bax was then calculated.

### 2.7. Statistical Analysis

SPSS 17.0 statistical software was used to analyze the data, and the results are presented as mean ± standard deviation ($\bar{x}\pm s$). Variance was applied to evaluate integral differences, and equal variance was compared between the two groups by means of* LSD*. A nonparametric test was used in the case of nonconformity with normal distribution. *P* < 0.05 was considered statistically significant.

## 3. Results

### 3.1. Observation of General Status

After modeling, the intake of food and water and defecation in the rats were normal. Infection and ulcers were not found on the distal limb. The rats walked by dragging toes or bouncing. Gait began to recover one week after treatment in the deep EA and shallow EA groups.

### 3.2. Changes in the S-D Curve in the Four Groups

**Figure 1:**
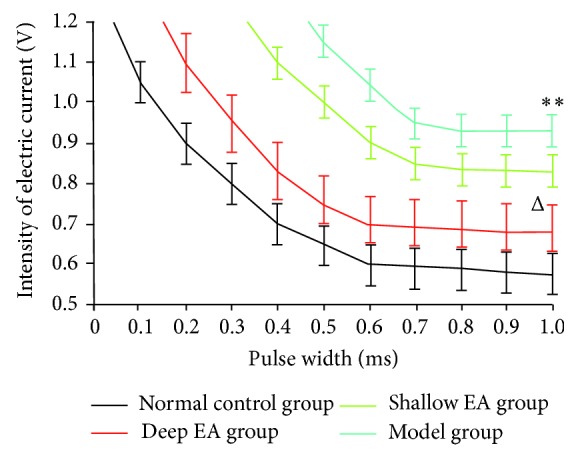
Effect of deep and shallow electroacupuncture (EA) stimulation of the ipsilateral “Huantiao” (GB 30) on the S-D curve in hip muscles of the rats with injured sciatic nerves ($\bar{x}\pm s$, 12 rats/group). ^**^ *P* < 0.01 versus the normal control group; ^Δ^ *P* < 0.05 versus the model group.

As shown in Figure 1, the upper segment of the S-D curve (0.1–0.5 ms) in each group was steeper, indicating that stimulus intensity decreased with prolonged stimulus time; the lower segment (0.6–1 ms) appeared flat, indicating that stimulus intensity remained constant when stimulus time was long. Under a certain stimulus time, the voltage which aroused excitability of the sciatic nerve in the four groups was the greatest in the model group, followed by the shallow EA group, the deep EA group, and the normal control group. Compared with the normal control group, the voltage in the model group increased significantly (*P* < 0.01); compared with the model group, the voltage reduced significantly in the deep EA group (*P* < 0.05), which showed that after two weeks of treatment, the best recovery of injured sciatic nerve was achieved in the deep EA group. The second best recovery of injured sciatic nerve occurred in the shallow EA group.

### 3.3. Changes in Conduction Velocity of Sciatic Nerves in the Four Groups

**Figure 2:**
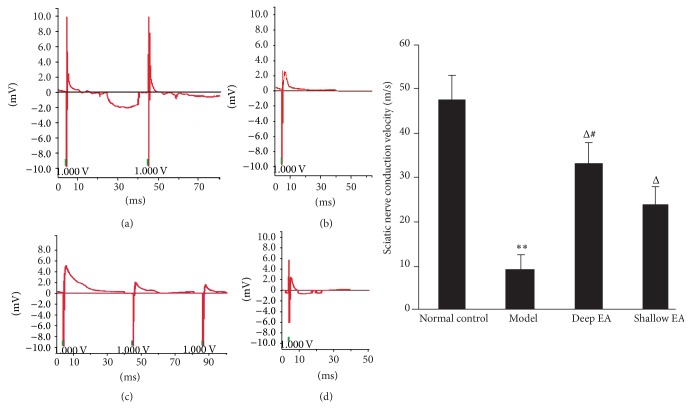
Effect of deep and shallow EA stimulation of ipsilateral GB 30 on the conduction velocity of the injured sciatic nerve in rats ($\bar{x}\pm s$, 12 rats/group). (a) Normal control group, (b) model group, (c) deep EA group, and (d) shallow EA group. ^**^ *P* < 0.01 versus the normal control group; ^Δ^ *P* < 0.05 versus the model group; ^#^ *P* < 0.05 versus the shallow EA group.

As shown in Figure 2, the conduction velocity in the model group decreased (*P* < 0.01), suggesting segmental demyelination of most nerve fibers; compared with the model group, the conduction velocity in the deep and shallow EA groups increased significantly (*P* < 0.05); the conduction velocity in the shallow EA group was markedly lower than that in the deep EA group (*P* < 0.05).

### 3.4. Changes in the Number of Apoptotic Cells in the Injured Sciatic Nerves in the Four Groups

**Figure 3:**
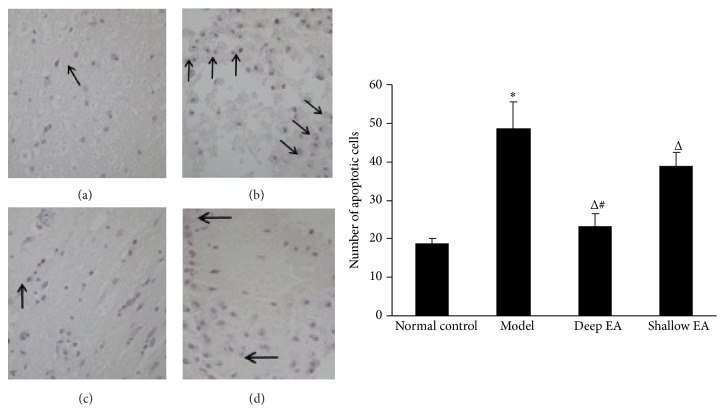
Effect of EA stimulation of ipsilateral GB 30 on number of apoptotic cells in the injured sciatic nerve in rats. Left panel: photos of TUNEL staining showing the number of apoptotic cells in the sciatic nerve (indicated by black arrowheads) in the normal control (a), model (b), deep EA (c), and shallow EA (d) groups (×200); right panel: bar graphs showing the number of apoptotic cells in the sciatic nerve in the four groups ($\bar{x}\pm s$, 8 rats/group). ^*^ *P* < 0.05 versus the normal control group; ^Δ^ *P* < 0.05 versus the model group; ^#^ *P* < 0.05 versus the shallow EA group.

As shown in Figure 3, the number of apoptotic cells in the model group was increased compared with that in the normal control group (*P* < 0.05), while the numbers of apoptotic cells in the deep EA and shallow EA groups were significantly decreased compared with those in the model group (*P* < 0.05). The effect of deep EA was better than that of shallow EA (*P* < 0.05).

### 3.5. Changes in the Expression of Bcl-2 and Bax in Injured Sciatic Nerves in the Four Groups

**Figure 4:**
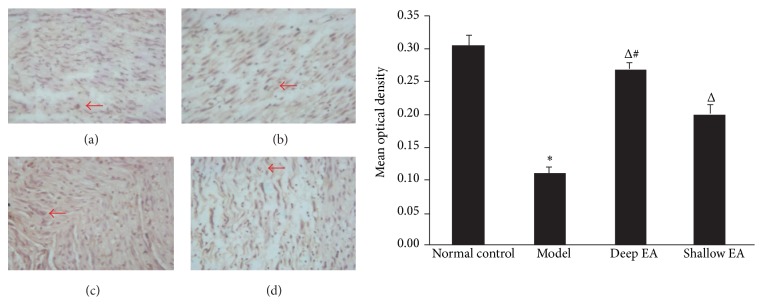
Effect of EA stimulation of ipsilateral GB 30 on Bcl-2 immunoreactivity in the injured sciatic nerve in rats. Left panel: photos of immunohistochemical staining showing the expression of Bcl-2 in the sciatic nerve (indicated by red arrowheads) in the normal control (a), model (b), deep EA (c), and shallow EA (d) groups (×200); right panel: bar graphs showing the expression levels (OD values) of Bcl-2 in the sciatic nerve in the four groups ($\bar{x}\pm s$, 8 rats/group). ^*^ *P* < 0.05 versus the normal control group; ^Δ^ *P* < 0.05 versus the model group; ^#^ *P* < 0.05 versus the shallow EA group.

**Figure 5:**
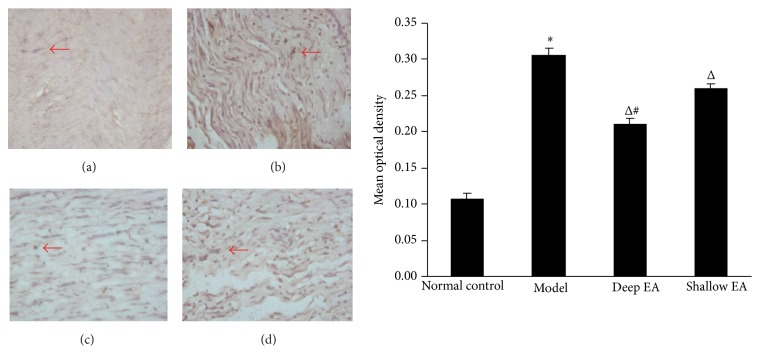
Effect of EA stimulation of ipsilateral GB 30 on Bax immunoreactivity in the injured sciatic nerve in rats. Left panel: photos of immunohistochemical staining showing the expression of Bax in the sciatic nerve (indicated by red arrowheads) in the normal control (a), model (b), deep EA (c), and shallow EA (d) groups (×200); right panel: bar graphs showing the expression levels (OD values) of Bax in the sciatic nerve in the four groups ($\bar{x}\pm s$, 8 rats/group). ^*^ *P* < 0.05 versus the normal control group; ^Δ^ *P* < 0.05 versus the model group; ^#^ *P* < 0.05 versus the shallow EA group.

As shown in Figures 4 and 5, the positive immunoreactivity of Bcl-2 and Bax proteins in the Schwann cell cytoplasm appeared brown. In the model group, the expression of Bcl-2 in sciatic nerve was significantly lower than that in the normal control group, while the expression of Bax was significantly higher (*P* < 0.05); compared with the model group, the expression of Bcl-2 increased and that of Bax decreased in the deep EA and shallow EA groups (*P* < 0.05). The difference between the deep EA group and the shallow EA group was statistically significant (*P* < 0.05).

### 3.6. Changes in the Ratio of Bcl-2/Bax in the Injured Sciatic Nerves in the Four Groups

**Figure 6:**
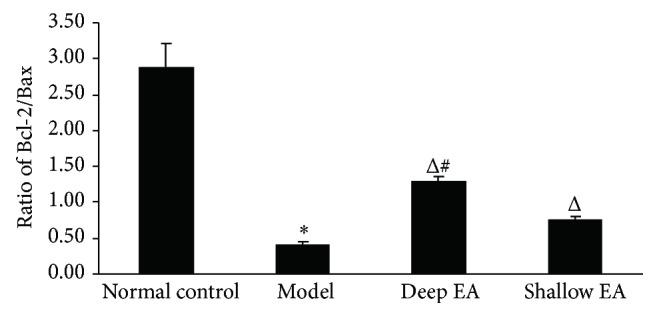
Effect of deep and shallow EA stimulation of ipsilateral GB 30 on the ratio of Bcl-2/Bax of the rats with injured sciatic nerves ($\bar{x}\pm s$, 8 rats/group). ^*^ *P* < 0.05 versus the normal control group; ^Δ^ *P* < 0.05 versus the model group; ^#^ *P* < 0.05 versus the shallow EA group.

As shown in Figure 6, the ratio of Bcl-2/Bax in the model group was markedly lower than that in the normal control group (*P* < 0.05), while the ratios in the deep EA and shallow EA groups were significantly higher than those in the model group (*P* < 0.05). The ratio in the deep EA group was higher than that in the shallow EA group (*P* < 0.05).

## 4. Discussion

This study was designed to investigate the biological mechanism involved in the differences in repair of sciatic nerve injury by deep EA and shallow EA at GB 30. The results showed that, compared with shallow EA, deep EA at GB 30, when the nerve trunk was reached, had a significantly better effect on the recovery of injured sciatic nerve. Deep EA improved the excitability of the nerve and promoted the recovery of motor nerve conduction, which may have been achieved by the increased expression of Bcl-2 and reduced expression of Bax, resulting in fewer apoptotic cells.

Bcl-2 gene families play important roles in apoptosis. According to their different roles, they are divided into two categories, one category can promote cell apoptosis; the other can inhibit cell apoptosis. Bcl-2 and Bax of the Bcl-2 gene families are two protein expression products closely related to apoptosis [14]. Bcl-2 is a type of membrane stabilizing protein related to organelles, especially mitochondria, and is mainly found in the outer membrane of mitochondria, endoplasmic reticulum membrane, and nuclear membrane. Inhibition of apoptosis can be achieved by inhibiting permeability of the mitochondrial membrane, maintaining the stability of the membrane, preventing the release of cytochrome C, and inhibiting the activation of Caspase by inhibition of free radical generation and intracellular calcium overload caused by Bcl-2. Cell damage can be reduced by the overexpression of Bcl-2 [15–17]. In contrast, the biological effect of Bax differs from that of Bcl-2 and can promote apoptosis [18, 19]. Bcl-2 and Bax may interact as a dimer. When Bcl-2 increases, the Bcl-2 homodimer is formed, and cells are protected; when Bax increases, the Bax/Bcl-2 heterodimer is formed, and cell apoptosis occurs [20–22]. Thus, the ratio of Bcl-2/Bax directly determines cell survival. The number of apoptotic cells is the balanced result of the two regulation factors, Bcl-2 and Bax [23]. It is generally recognized that the main form of neuronal cell death caused by peripheral nerve injury is apoptosis, a process of programmed cell death involving a variety of physiological and pathological factors and initiated by apoptosis-related genes [24–26]. Kotulska et al. [27] suggested that the expression of Bcl-2 and Bax is closely related to the recovery and viability of neurons after peripheral nerve injury as well as fiber regeneration and myelination. In this study, the function of the sciatic nerve in rats in each group varied due to changes in Bcl-2 and Bax at the injured site, which is consistent with previous results.

The S-D curve is used to evaluate the function of innervation by stimulating muscle with a square wave current of different pulse width. It is reflected by the curve of threshold of current strength when the muscle is excited. The lighter the nerve injury, the less denervation, and the lower the S-D curve, the lower the stimulus intensity to cause nerve-muscle excitation. The results of this study showed that in the deep EA group, the current intensity to induce nerve excitement was markedly lower than that in the model group, indicating better efficacy of deep EA in promoting the recovery of injured sciatic nerve. As a direct manifestation of nerve impulse conduction, the conduction velocity can reflect the regeneration conditions of nerve fibers to some extent. The nerve trunk cannot be completely destroyed by clamping sciatic nerves. Therefore, the rats, in which nerve conduction was completely blocked and the electric waves could not be displayed, were excluded from this study.

The treatment of flaccidity syndrome by acupuncture at GB 30 can be traced back to the* Internal Canon of Medicine*. The depth of acupuncture may determine its clinical efficacy [28, 29]. In recent years, the depth of acupuncture at GB 30 has been increased. It is proposed that 2-3 cun is appropriate to ensure that the nerve trunk is reached [30–32]. Studies have shown that deep acupuncture at GB 30 may have immediate and remarkable therapeutic and analgesic effects on sciatic nerve injury [33]. Research on the related mechanisms suggests that, for sciatic nerve injury in rabbits, acupuncture therapy can increase the content of acetylcholine esterase (AchE) in the intumescentia lumbalis of the spinal cord. By promoting the synthesis and release of AchE, nerve excitability was maintained thus promoting the recovery of nerve function [34]. EA can increase the content of 1L-1*β* in spinal cord tissue and stimulate the production of nerve growth factors (NGFs) [35], thus strengthening the transcription of NGF mRNA and exerting a positive effect on the repair of injured sciatic nerves [36]. The results of this study showed that, compared with shallow EA, deep EA at GB 30 better adjusted the substances related to the regulation of apoptosis, thus significantly improved the excitability and conduction of the sciatic nerve.

## 5. Conclusion

Better functional recovery of injured sciatic nerve may be achieved by acupuncture at GB 30 when the nerve trunk is reached. By stimulating the axis cylinder with continuous and chronic demyelination, strong stimulation is generated, triggering strong nerve impulses to transfer nerve substances, which can inhibit the apoptosis of nerve cells in the injured area and promote tissue repair.
